# Disparities in Non-Fatal Health Outcomes in Pediatric General Trauma Studies

**DOI:** 10.3390/ijerph15010043

**Published:** 2017-12-27

**Authors:** Shanthi Ameratunga, Jacqueline Ramke, Nicki Jackson, Sandar Tin Tin, Belinda Gabbe

**Affiliations:** 1 Epidemiology and Biostatistics, School of Population Health, The University of Auckland, Private Bag 92019, Auckland 1142, New Zealand; j.ramke@auckland.ac.nz (J.R.); s.tintin@auckland.ac.nz (S.T.T.); 2 Alcohol Healthwatch, Office Park Building Level 1, 27 Gillies Ave, Auckland 1023, New Zealand; director@ahw.org.nz; 3 Department of Epidemiology and Preventive Medicine, Monash University, School of Public Health and Preventive Medicine, Level 3, 553 St Kilda Road, Melbourne 3004, Australia; belinda.gabbe@monash.edu; 4 Farr Institute, Swansea University Medical School, Swansea University, Swansea SA2 8PP, UK

**Keywords:** injury, children, prognosis, disability, quality of life, functional outcomes, health inequalities, disparities, socio-economic, ethnicity/race

## Abstract

When prevention efforts fail, injured children require high-quality health services to support their recovery. Disparities in non-fatal injury outcomes, an indicator of health-care quality, have received minimal attention. We evaluated the extent to which general trauma follow-up studies published in the peer-reviewed scientific literature provide evidence of socially patterned inequities in health, functional or disability outcomes ≥4 weeks after childhood injuries. Using a systematic search, we identified 27 eligible cohort studies from 13 high-income countries. We examined the extent to which the reported health outcomes varied across the PROGRESS criteria: place of residence, race/ethnicity, occupation, gender/sex, religion, socio-economic status, and social capital. The available evidence on differential outcomes is limited as many studies were compromised by selection or retention biases that reduced the participation of children from demographic groups at increased risk of adverse outcomes, or the analyses mainly focused on variations in outcomes by sex. Given the limited research evidence, we recommend greater attention to systematic collection and reporting of non-fatal injury outcomes disaggregated by socio-demographic indicators in order to identify disparities where these exist and inform equity-focused interventions promoting the recovery of injured children.

## 1. Introduction

Globally 950,000 children aged <18 years die from injuries each year and 10–30 million experience non-fatal injuries [1]. This burden is disproportionately greater in low- and middle-income countries, and recent evidence suggests that disparities in child injury deaths are worsening in some contexts, including within high-income countries [2]. Reported disparities in child injury deaths are particularly likely by ethnicity/race, socio-economic status and insurance cover [3,4,5,6,7]. In contrast, the extent to which non-fatal outcomes (e.g., longer-term disability) following childhood injury may vary by sociodemographic factors has received little attention.

Acknowledging non-fatal childhood injuries as a neglected public health problem with substantial health, social, and economic costs [8,9,10], the 64th World Health Assembly adopted a child injury prevention resolution calling on member states to strengthen emergency and rehabilitation services in order to improve outcomes for injured or disabled children [11]. However, well-recognized situations with other health conditions in addition to the trends in child injury deaths noted above raise the possibility that even when improvements in non-fatal injury outcomes and recovery trajectories are realized at the population level, there could be intractable or widening inequalities between sub-groups [2,12]. Factors ranging from increased exposure to injuries to unequal access to high-quality emergency trauma and rehabilitation care can place children in socially disadvantaged groups at increased risk of injury-related disability [13,14,15,16,17,18].

A recent systematic review and meta-analysis undertaken by Haider et al. found strong evidence for disparate trauma outcomes by race/ethnicity and insurance status in the United States [19]. However, few studies in this review focused on children, and these were limited to mortality or discharge from emergency departments as an outcome, or traumatic brain injury as the mechanism of injury. While no equivalent review has been undertaken at a global level, the findings suggest a gap in necessary knowledge, not only in terms of evidence of the presence (or not) of disparities, but also in primary research studies investigating the possibility of such disparities. Not surprisingly, evaluations of interventions designed to reduce inequities in trauma outcomes by race/ethnicity or socio-economic status are also sparse [20].

The Sustainable Development Goals adopted unanimously by the 193 member states of the United Nations embrace the principle of ‘leaving no one behind’. This laudable goal demands systematic enquiries that can identify groups more vulnerable to adverse health outcomes and equity-focused interventions that can reduce these disparities. It is, therefore, important to consider the extent to which current research evidence supports this agenda. In doing so, we have to acknowledge the many methodological issues that can challenge the conduct of informative research in this field. Recruiting and prospectively following heterogeneous samples of injured children over time can be resource-intensive and fraught with problems resulting in participants being lost to follow-up. By design or practice, studies could exclude some groups, or have inadequate explanatory power to investigate differential outcomes between sub-groups within the sample.

We aimed to systematically search the published epidemiological research investigating non-fatal outcomes among injured children, and summarize the extent to which these studies consider or report research participation and outcomes by social sub-groups. To achieve this aim, we used the ‘PROGRESS’ framework which provides a systematic approach to identifying axes of social stratification linked to health inequality: Place of residence; Race/ethnicity/culture/language; Occupation; Gender/sex; Religion; Education; Socio-economic status; Social capital [21].

## 2. Materials and Methods 

We drew on published guidelines on conducting [22,23] and reporting [24] systematic reviews that consider health equity.

### 2.1. Search Strategy

We sought evidence of disability or general health outcomes following non-fatal injuries among children and adolescents (aged <18 years). We purposely constructed a broad search focusing on these outcomes to assess the extent to which studies reporting these outcomes consider equity. Studies could be undertaken in any country and published in any language from 1990 to 30 June 2017.

The search was constructed in consultation with a medical librarian. On 17 July 2017 we searched Medline, Embase and Web of Science for studies published between January 1990 and June 2017. The Medline search strategy is included as the Supplementary Materials (File S1). To identify additional studies, reference lists of systematic reviews [4,25,26,27,28,29] identified in the search were examined. References of identified articles were also searched for additional relevant articles. Articles were imported into EndNote (Thompson Reuters, San Francisco, CA, USA) and duplicates removed.

### 2.2. Study Selection

Selection criteria were developed by the authors at the start of this review. Then, two reviewers (J.R. and one of S.A., S.T.T. or B.G.) independently screened the titles of all citations identified during the initial search and the full-text manuscript was retrieved for review to determine if the citation was potentially relevant. Study selection was finalized by discussion between S.A. and J.R.

Studies were included if they were cohort studies, recruited participants after 1 January 1990, and reported at least one disability or general health outcome beyond the acute phase (i.e., at least 4 weeks after the injury). Studies that included both adult and child/adolescent participants were included if separate analyses were reported for the group younger than 18 years.

Studies were included if these related to health outcomes of general trauma populations that were heterogeneous with regard to injury diagnostic categories, regardless of the mechanism of injury. Studies were excluded if they were restricted to patients with a specific injury diagnostic category (e.g., traumatic brain injury, spinal cord injury, pelvic fractures, submersion injuries, whiplash injuries, burns, or poisoning); or if they reported a specific mental health outcome (e.g., post-traumatic stress disorder) without also reporting a functional disability outcome or general health status measure.

### 2.3. Data Extraction and Synthesis

A data extraction form was created and pilot-tested on a subset of six included studies to ensure complete and appropriate information was extracted. The updated form was used by one reviewer (J.R.) to extract data, and these extracted data were verified by another reviewer (N.J.). From each included study the following information was extracted: lead author, year of publication, country of study, recruitment period, sample size, recruitment and follow-up rates, length of follow-up, and the tool used to measure the disability or health outcome.

To determine the extent to which disparities were considered in the included studies we recorded whether PROGRESS dimensions [21] were reported and/or analyzed for each study, particularly in relation to: (i) inclusion/exclusion criteria; (ii) loss to follow-up; (iii) participant characteristics; and (iv) health or disability outcomes.

We also hypothesized that disadvantaged children were more likely to be under-represented in injury follow-up studies. To examine this possibility, we extracted data on losses to follow-up and the proportions of the eligible populations that were assessed at the final follow-up. These features were determined to be the most relevant items for our review from among the items incorporated in the National Institutes of Health quality-assessment tool for cohort studies [30].

We used descriptive analysis and narrative synthesis to summarize the ways in which inequality was reported and analyzed in the included studies.

## 3. Results

### 3.1. Summary of Included Studies

**Figure 1:**
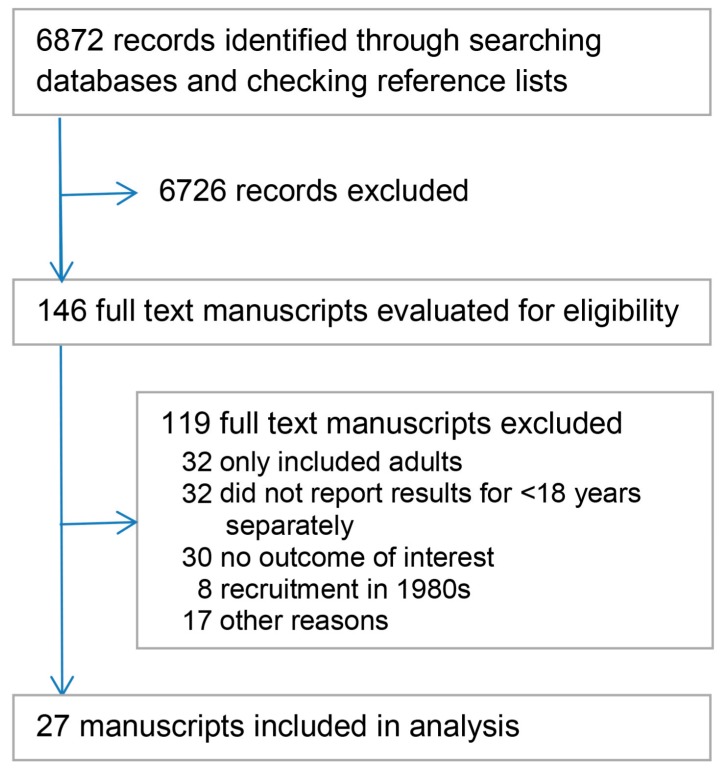
Summary of study search and selection.

Of the 6872 studies identified in the search, 27 met the review inclusion criteria. Search and study selection is summarized in Figure 1 and the setting and characteristics of included studies are summarized in Table 1. Table 2 provides the specific information linked to each study. Most studies (*n* = 25) were published in English, with one each in French and Spanish. The studies were undertaken in 13 high-income countries (the most common being The Netherlands *n* = 6 and USA *n* = 4). Cohorts were commonly recruited from hospitals, either while the child was admitted, or from a register of injured children. The median number of participants at the final follow-up was 146 (inter-quartile range (IQR) 107–241) and the median final follow-up rate was 64% (IQR 57–73%). The most common follow-up period was between 6 and 12 months after the injury, and the most frequently used method to determine the outcome was a bespoke question rather than a validated tool (Table 1).

### 3.2. PROGRESS Dimensions in Eligibility and Participation

Eight studies (30%) required participants to be able to communicate in a specific language as a cohort inclusion/exclusion criterion, but only one of these studies reflected on the implication of the exclusion. This study from Switzerland recognized that eligible families were more likely to be of higher socio-economic status (SES) than those excluded because an inability to speak German was more likely among non-Swiss nationals who tended to have lower SES [50].

Ten studies (37%) included at least one PROGRESS dimension when reporting and assessing loss to follow-up (Table 3). As summarized in Table 4, only one study (of nine studies assessing variations by sex) found a difference with girls more likely to be lost to follow-up [52]. The one study examining parental occupation found higher losses to follow-up among children of parents with low occupation level [6]. The study assessing socio-economic status found loss to follow-up was highest among those in the lowest income quintile [43]. The one study assessing variations by nationality found no difference in loss to follow-up between Swiss/German and other children [50].

Most studies (*n* = 25; 93%) reported participant characteristics by at least one PROGRESS dimension (details in Table 3). All dimensions were reported in at least one study, except for religion. Sex was most commonly reported (*n* = 24), followed by race/ethnicity/culture/language (*n* = 7).

### 3.3. Reporting of Outcomes

Ten studies (37%) reported results disaggregated by at least one PROGRESS dimension, four of which found significant differential outcomes in unadjusted models (Table 4). Eight studies (30%) disaggregated the outcome by sex, seven of these tested for a difference between girls and boys and two found that girls had worse outcomes than boys [38,42]. The one study that assessed outcomes by education level found that children with parents in the lowest education level had worse outcomes than children with more educated parents [39]. Socio-economic status was assessed in two studies while race/ethnicity and occupation were assessed by one study each with no difference in outcomes found. One [36] of the two studies assessing place of residence found differences in outcomes in study reporting centres in the UK (Bristol, Nottingham, Surrey and Swansea), the underlying reasons for which were not self-evident. None of the studies in our review comment on testing for interactions in relation to general health outcomes. However, one study by Sturm et al. [47] implies this in the Methods section of the study but the findings from tests of interaction are reported only in relation to the Impact of Events (post-traumatic stress) data, finding no differences in outcomes by age, gender, or socio-economic status.

Thirteen studies (48%) considered at least one PROGRESS dimension when investigating risk factors for adverse health or disability outcome, with four studies reporting significant findings in adjusted models (Table 4). Again, sex was the factor investigated and reported most often (*n* = 11), with other PROGRESS dimensions included in no more than two studies. Girls were found to be at higher risk of poor outcomes in three studies [34,36,40]. Place of residence [36] and low parental education level [46] were found to be predictors of worse outcomes in some contexts. None of the very few studies examining the possibility found significant associations with race/ethnicity/nationality, socio-economic status or parental marital status (as a proxy for social capital).

## 4. Discussion

Understanding the nature and extent of disparities in non-fatal outcomes following childhood injuries is essential for informing strategies to overcome them. Our review found that most follow-up studies of general trauma in childhood missed opportunities to obtain evidence that can inform equitable high-quality trauma and rehabilitation care, suggesting oversights with ethical and social justice implications. Socially disadvantaged injured children were under-represented in many studies due to their lesser likelihood of being invited to participate, consenting to participate, or being retained in studies over time. With the exception of variations by gender, investigations of differential outcomes by PROGRESS dimensions were infrequent. When disadvantaged groups are less likely to be study participants or their lived realities are masked in analyses, the potential to uncover and understand disparities is reduced [33] and the perennial problem of ‘no data, no problem’ looms large [53].

Previous research involving pediatric trauma patients reveals that families less likely to participate in research are often more likely to need particular attention and context-specific responses to enable equitable outcomes [51]. Disproportionately higher losses to follow-up of socially disadvantaged groups in research can lead to under-estimation of socio-economic inequalities in health outcomes [54]. In a clinical parallel, the primary reasons for not attending post-discharge clinic appointments by respondents in a trauma registry-based study from Indiana, USA [55], were directly linked to social disadvantage, e.g., financial difficulties, travel distance, and lack of a support person who could assist in getting to the clinic. Using a large trauma database, Leukhardt et al. [56] identified a range of socio-demographic factors associated with disparities in follow-up after trauma, and highlighted deficiencies in medical charts that undermined approaches to address these.

The apparent absence of research on inequities in non-fatal outcomes following child injury in low- and middle-income country settings highlights a major gap in current evidence as over 95% of child injury deaths occur in these settings [1]. Furthermore, those who survive injuries experience multiple barriers accessing health and rehabilitation services, especially those who are poor, live in rural settings, or belong to disadvantaged ethnic, racial or indigenous communities [57].

While this study was undertaken by experienced reviewers implementing a systematic approach to searching, selecting studies and extracting data from peer-reviewed scientific journal articles, our analysis must be interpreted in light of several limitations. We did not pre-publish a review protocol and did not search the ‘grey’ literature (e.g., conference proceedings, dissertations). We did not impose a language restriction; publication bias can result in research in non-English speaking settings or low- and middle-income countries being less likely to be identified.

Given our focus on general trauma populations and the review exclusions applied, we cannot determine if follow-up studies of patients with specific injury diagnostic categories such as burns or poisoning are more or less likely to report variations in non-fatal health outcomes by PROGRESS criteria. It is also important to acknowledge that axes of difference other than PROGRESS criteria can lead to disparities in health outcomes, e.g., migrant status, pre-existing disability, sexual identity, experience of racism and discrimination.

Importantly, this review focused only on patient characteristics. As noted in the Institute of Medicine Report on ethnic and racial disparities in health care, provider factors such as implicit (or unconscious) biases can influence clinical decisions relating to differential approaches to treatment, referrals, and patient-provider relationships [16,18]. Unequal outcomes can also result from systemic, organisational and structural aspects of the health-care system, including unreasonable demands on health literacy, challenging clinical pathways, overcrowding and access block at emergency departments, and competing demands on services [12,19,58,59]. Haider et al. [18] use available literature to contextualise the likely contributors to disparities across the continuum of trauma care, mediated by race, insurance status and socio-economic status. They propose an overarching framework to examine disparities with clusters of important influences relating to the injured child (host), and pre-hospital, hospital/provider, post-acute care and rehabilitation contexts. More nuanced analyses can also uncover possible interactions between different factors. For example, a Californian study which found no racial/ethnic disparity in mortality for injured children suggested that the unexpected finding was possibly due to insurance coverage being less of a barrier for children in California than for adults and children elsewhere in the United Sates, and because California’s racial diversity led to culturally competent care which delivered better outcomes [6].

Arguably, sub-group analyses across PROGRESS dimensions can introduce risks of multiple comparisons that could be considered statistical malpractice [35]. In pursuit of health equity and ‘leaving no one behind’, a solution needs to be found to enable consideration of differential outcomes across relevant PROGRESS dimensions. Approaches to consider include equivalents to the guidelines for sub-group analysis in randomized controlled trials and meta-analyses [35,49] and mediation analysis, where the focus is on identifying the contribution of a range of factors that influence differential health outcomes across population sub-groups (e.g., ethnic groups, geographic locations, different health-financing systems, or stages of illness) [60].

The issues highlighted in this review inevitably overlap with the probability that health-care interventions can have differential impacts. It is reasonably argued that a key requisite for improving outcomes following pediatric trauma is knowing “what works, for who, and in what circumstances” [34]. When undertaking observational studies, clinical trials and mixed-methods research that can inform these questions [34,61], it is also important to consider underlying factors that could result in unequal treatment outcomes [19,20]. A comprehensive summit on surgical disparities convened by the National Institutes of Health and the American College of Surgeons identified the need to prioritize research on the perspectives of patients regarding the care they received, the diversity and training of the workforce, and technologies that could mitigate disparities [59]. Drawing from our review, we would add the need to consider the ethical principles, governance, design, analysis and dissemination of findings that ensure experiences of socially disadvantaged children are explicitly identified and addressed.

## 5. Conclusions

Based on our review, there is insufficient evidence to determine the extent to which recovery trajectories of injured children are patterned by underlying social factors. Given the burden of health inequities borne by children from disadvantaged groups, research investigating the health of injury survivors should explicitly examine the potential for disparate outcomes. Similarly, evaluations of health-care interventions for injured children should investigate if these do not unwittingly increase inequity.

## Figures and Tables

**Table 1:** Summary of characteristics of included studies.

Study Characteristics	Details
Country	Number of studies
The Netherlands	6
USA	4
Canada	3
Australia, France, Israel, Switzerland	2 each
Austria, Belgium, Croatia, Spain, Sweden, UK	1 each
Number of participants at final follow-up *	Number of participants
Median (inter-quartile range)	146 (107–241)
Range	28–700
Proportion of eligible children in final follow-up *	%
Median (inter-quartile range)	64 (57–73)
Range	20–90
Period of final follow-up (years since injury)	Number of studies
≤0.5	7
>0.5 ≤ 1	10
>1 ≤ 3	4
>3	6
Tool used to measure outcome **	Number of studies
A study-specific question/questionnaire	8
TACQOL	5
Child Health Questionnaire (CHQ)-PF50/PF28/CF87	5
FIM/WeeFIM	4
Glasgow Outcome Scale/GOS-Extended	3
PEDSQL	2
EQ-5D, FS-II, IROS, KOSCHI, QWB, RAHC MOF, Rand Health Insurance, VABS, CBCL, SDQ, POPC	1 each

* Cannot determine in 5 studies; ** adds up to >27 as some studies used >1 tool. Abbreviations: TACQOL = Toegepast Natuurwetenschappelijk Onderzoek-Academisch Ziekenhuis Leiden (TNO-AZL) Children’s Quality of Life; CHQ = Child Health Questionnaire; PF = parent form; CF = child form; FIM = Functional Independence Measure; GOSE = Glasgow Outcome Scale Extended; PEDSQL = Pediatric Quality of Life Inventory; EQ-5D = EuroQoL – 5 Dimensions; FS-II = Functional Status II; IROS = ICF (International Classification of Functioning)-Related Outcome Score; KOSCHI = King’s Outcome Scale for Childhood Head Injury; QWB = Quality of Well Being; RAHC MOF = Royal Alexandra Hospital for Children Measure of Function; VABS = Vineland Adaptive Behaviour Scale; CBCL = Child Behaviour Checklist; SDQ = Strengths and Difficulties Questionnaire; POPC = Pediatric Overall Performance Category.

**Table 2:** Specific characteristics of included studies including setting, measurement tools and follow-up features.

Study (Alphabetical Order)	Country	Number of Children at Final Follow-Up	Proportion of Eligible Children Included at Final Follow-Up (%)	Period of Final Follow-Up (Years Since Injury)	Tool Used to Measure Outcome **
Aitken 2002 [3]	USA	141	45.5	0.5	CHQ-PF50, FIM/WeeFIM
Batailler 2014 [6]	France	127	*	1	Study-specific question(s)
Davey 2005 [7]	Australia	241	57.4	1–2	CHQ-PF50
Dekker 2004 [31]	The Netherlands	100	71.9	2–7	CHQ-CF87
Gabbe 2011 [32]	Australia	144	*	1	FIM, GOS, KOSCHI, CHQ-PF28, PEDSQL
Gofin 1999 [33]	Israel	not reported	*	0.5	for 4–17 years 25 items questionnaire developed from International Classification of Impairments, Disabilities and Handicaps; study-specific questions for 0–3 years age group
Gofin 2007 [5]	Israel	549	59.4	0.42	Study-specific question(s)
Holbrook 2007 [34]	USA	356	88.8	2	Quality of wellbeing scale (QWB)
Janssens 2009 [35]	The Netherlands	28	70.0	6–8	GOS, GOSE, VABS, CBCL, SDQ
Kendrick 2013 [36]	UK	164	*	1	Study-specific question(s)
Landolt 2009 [9]	Switzerland	68	58.6	1	TACQOL + study-specific question(s)
Macpherson 2003 [37]	Canada	357	73.0	0.5	WeeFIM
Mestrovic 2013 [38]	Croatia	not reported	*	1	RAHC MOF to assess HRQOL
Oloffson 2012 [39]	Sweden	341	81.2	1–1.67	Study-specific question(s)
Polinder 2005 [40]	The Netherlands	365	29.9	0.75	EuroQol (EQ-5D)
Pumar 2007 [41]	Spain	209	*	2–5	POPC
Schalamon 2003 [42]	Austria	58	82.9	2–9	GOS
Schneeberg 2017 [43]	Canada	161	19.5	1	PEDSQL
Schweer 2006 [44]	USA	128	22.2	0.5	CHQ-PF50
Sturms 2002a [45]	The Netherlands	59	67.8	0.5–2.1	FS-II, TACQOL
Sturms 2002b [46]	The Netherlands	211	64.5	1.5–3.4	TACQOL
Sturms 2005 [47]	The Netherlands	51	35.7	0.5	TACQOL
Valadka 2000 [48]	Canada	116	58.3	not reported	Rand Health Insurance Study physical health scales + study question(s)
van de Voorde 2011 [49]	Belgium	146	63.8	1	IROS
Vollrath 2005 [50]	Switzerland	107	60.1	1	TACQOL
Winthrop 2005 [51]	USA	156	86.7	0.5	FIM/WeeFIM
Yacoubovitch 1995 [52]	France	700	*	1	Study-specific question(s)

* Numerator or denominator not provided, ** Common abbreviations for named tools noted in full at the foot of Table 1.

**Table 3:** Frequency of studies providing data on PROGRESS dimensions.

Social Dimension	Baseline Participant Characteristics	Loss to Follow-Up		Disaggregated/Unadjusted Outcomes		Adjusted Outcomes	
	Reported *n* (%)	Reported *n* (%)	Difference Found *	Reported *n* (%)	Difference Found *	Reported *n* (%)	Difference Found *
P Place of residence	1 (4)	-	-	2 (7)	1	1 (4)	1
R Race/ethnicity/culture/language	7 (26)	1 (4)	-	2 (7)	-	2 (7)	-
O Occupation	2 (7)	1 (4)	1	1 (4)	-	-	-
G Gender/sex	24 (89)	9 (33)	1	8 (30)	2	11 (41)	3
R Religion	-	-	-	-	-	-	-
E Education	1 (4)	-	-	1 (4)	1	2 (7)	1
S Socio-economic status	4 (15)	1 (4)	1	2 (7)	-	1 (4)	-
S Social capital	2 (7)	-	-	-	-	1 (4)	-
**At least one**	25 (93)	10 (37)		10 (37)		13 (48)	

* Reported differences between sub-groups are summarized in Table 4.

**Table 4:** General trauma follow-up studies in childhood identifying influences on, or significant variations in, health outcomes by PROGRESS criteria.

Explanatory Factor	PROGRESS Criteria	Study	Reported Difference(s) in Children	
Propensity of being lost to follow-up (a key factor that could influence investigations of predictors of differential health outcomes)	Occupation	Batailler 2014 [6]	Non-respondents were reported as more likely to be of lower socio-economic occupational level; no related data or statistics provided	
	Gender	Yacoubovitch 1995 [52]	8% of boys and 13% of girls were lost to follow-up; no statistics provided	
	Socio-economic status (SES)	Schneeberg 2017 [43]	Participants in low-income families less available for 12 months follow-up interview. Odds Ratio (95% CI)	
			5 (highest-income quintile)	1.0 (reference)
			4	0.5 (0.2–1.4)
			3	1.8 (0.6–4.9)
			2	0.7 (0.3–1.8)
			1 (lowest income quintile)	0.3 (0.1–0.7)
Unadjusted outcomes	Place	Kendrick 2013 [36]	Variations in recovery at 12 months by study site Relative Risk (95% CI)	
			Nottingham	1.00
			Bristol	1.17 (0.98–1.41)
			Swansea	1.22 (1.05–1.40)
			Surrey	1.21 (1.05–1.39)
	Gender	Yacoubovitch 1995 [52]	20% girls compared with 12% boys had not recovered at 12 months (*p* < 0.01)	
		Polinder 2005 [40]	Girls more likely than boys to have sub-optimal functioning at 12 months Odds Ratio (95% CI)	
			Boy	1.0
			Girl	2.9 (1.0–9.9)
	Education	Sturms 2002b [46]	Predictors of lower health-related quality of life at a mean follow-up period of 2.4 years post-injury	
			Lower educational level of father (*p* = 0.001)	
			Lower educational level of mother (*p* < 0.001)	
Adjusted outcomes	Place	Kendrick 2013 [36]	Recovery at 12 months: Relative Risk (95% CI)	
			Nottingham	1.00
			Bristol	1.01 (0.88–1.16)
			Swansea	1.12 (0.99–1.27)
			Surrey	1.15 (1.02–1.30)
	Gender	Kendrick 2013 [36]	Recovery at 12 months: Relative Risk (95% CI)	
			Girls	1.0
			Boys	1.15 (1.03–1.27)
		Polinder 2005 [40]	Sub-optimal functioning at 12 months Odds Ratio (95% CI)	
			Boy	1.0
			Girl	3.0 (1.0–11.0)
		Holbrook 2007 [34]	Results section notes the Quality of Well Being scores at 18-month follow-up were lower in adolescent girls than in boys; no data provided	
	Education	Sturms 2002b [46]	Lower education of fathers and mothers (highly correlated) were significant explanatory variables of lower health-related quality of life of children. Estimate for mothers’ educational level provided (*p* < 0.001)	

## Supplementary Materials:

The following are available online at www.mdpi.com/1660-4601/15/1/43/s1, File S1: Medline search strategy, File S2: List of included studies.
