# Supplementary material for: Disparities in Non-Fatal Health Outcomes in Pediatric General Trauma Studies
Source: Int J Environ Res Public Health. 2017 Dec 27;15(1):43. doi: 10.3390/ijerph15010043 (PMC5800142; doi:10.3390/ijerph15010043)
Supplement: Supplementary file 1 [file ijerph-15-00043-s001.pdf]

**Supplementary File 1: Medline search strategy**

1. accidents/ or accidental falls/ or accidents, home/ or accidents, traffic/ or drowning/ or near drowning/
2. "Wounds and Injuries"/
3. unintentional*.mp. [mp=title, abstract, original title, name of substance word, subject heading word, keyword heading word, protocol supplementary concept word, rare disease supplementary concept word, unique identifier]
4. 2 and 3
5. accident*.mp. [mp=title, abstract, original title, name of substance word, subject heading word, keyword heading word, protocol supplementary concept word, rare disease supplementary concept word, unique identifier]
6. 1 or 4 or 5
7. Disability Evaluation/
8. "International Classification of Functioning, Disability and Health"/
9. "outcome and process assessment (health care)"/ or "outcome assessment (health care)"/ or patient outcome assessment/ or critical care outcomes/ or treatment outcome/ or "process assessment (health care)"/
10. "Recovery of Function"/
11. "Quality of Life"/
12. "Activities of Daily Living"/
13. ((physical* or functional*) adj (impair* or disab* or status)).mp. [mp=title, abstract, original title, name of substance word, subject heading word, keyword heading word, protocol supplementary concept word, rare disease supplementary concept word, unique identifier]
14. "quality of life".mp. [mp=title, abstract, original title, name of substance word, subject heading word, keyword heading word, protocol supplementary concept word, rare disease supplementary concept word, unique identifier]
15. 13 or 14
16. 7 or 8 or 9 or 10 or 11 or 12 or 15
17. 6 and 16
18. limit 17 to "all child (0 to 18 years)"
19. limit 18 to yr="1990 -Current"
20. limit 19 to english language
21. 19 not 20

**Supplementary File 2: Included studies**

Aitken M, Tilford J, Barrett K, et al. Health status of children after admission for injury. Pediatrics. 2002;110:337-42.

Batailler P, Hours M, Maza M, et al. Health status recovery at one year in children injured in a road accident: A cohort study. Accid Anal Prev 2014;71:267-72.

Davey TM, Aitken LM, Kassulke D, et al. Long-term outcomes of seriously injured children: A study using the Child Health Questionnaire. J Paediatr Child Health. 2005;41:278-83.

Dekker R, van der Sluis CK, Kootstra J, et al. Long-term outcome of equestrian injuries in children. Disabil Rehabil. 2004;26:91-96.

Gabbe B, Simpson P, Sutherland A, et al. Functional and health-related quality of life outcomes after pediatric trauma. J Trauma. 2011;70:1532-38.

Gofin R, Adler B, Hass T. Incidence and impact of childhood and adolescent injuries: a population-based study. J Trauma Acute Care Surg. 1999;47:15-21.

Gofin R, Avitzour M. Outcome of head and other injuries among Israeli children: physical limitations and stress symptoms. Isr Med Assoc J. 2007;9:531-36.

Holbrook TL, Hoyt DB, Coimbra R, et al. Trauma in adolescents causes long-term marked deficits in quality of life: adolescent children do not recover preinjury quality of life or function up to two years postinjury compared to national norms. J Trauma Acute Care Surg. 2007;62:577-83.

Janssens L, Willem Gorter J, Ketelaar M, et al. Long-term health condition in major pediatric trauma: a pilot study. J Pediatr Surg. 2009;44:1591-600.

Kendrick D, Vinogradova Y, Coupland C, et al. Recovery from injury: the UK burden of injury multicentre longitudinal study. Inj Prev. 2013;19:370-81.

Landolt MA, Vollrath ME, Gnehm HE, et al. Post-traumatic stress impacts on quality of life in children after road traffic accidents: Prospective study. Aust N Z J Psychiatry. 2009;43:746-53.

Macpherson A, Rothman L, McKeag A, et al. Mechanism of injury affects 6-month functional outcome in children hospitalized because of severe injury. J Trauma. 2003;55:454-58.

Meštrović J, Meštrović M, Polić B, et al. Clinical scoring systems in predicting health-related quality of life of children with injuries. Coll Antropol. 2013;37:373-77.

Olofsson E, Bunketorp O, Andersson A-L. Children at risk of residual physical problems after public road traffic injuries—A 1-year follow-up study. Injury. 2012;43:84-90.

Polinder S, Meerding WJ, Toet H, et al. Prevalence and prognostic factors of disability after childhood injury. Pediatrics. 2005;116:e810-e17.

Rivas Pumar P, Rodriguez Nunez A, Blanco-Ons Fernandez P, et al. Consecuencias a largo plazo de los traumatismos pediatricos que precisaron cuidados intensivos. *An Pediatr (Barc)*. 2007;66:4-10.

Schalamon J, Bismarck Sv, Schober PH, et al. Multiple trauma in pediatric patients. Pediatr Surg Int. 2003;19:417-23.

Schneeberg A, Ishikawa T, Kruse S, et al. A longitudinal study on quality of life after injury in children. Health Qual Life Outcomes. 2016;14:120.

Schweer LH, Cook BS, Bivens K, et al. Family perception: quality of life following a child's traumatic injury. J Trauma Nurs. 2006;13:6-14.

Sturms LM, van der Sluis CK, Groothoff JW, et al. The health-related quality of life of pediatric traffic victims. J Trauma Acute Care Surg. 2002;52:88-94.

Sturms L. Pediatric traffic injuries: consequences for the child and the parents. PhD Thesis. University of Groningen, the Netherlands, 2002.

Sturms L, van der Sluis CK, Stewart RE, et al. A prospective study on paediatric traffic injuries: health-related quality of life and post-traumatic stress. Clin Rehabil. 2005;19:312-22.

Valadka S, Poenaru D, Dueck A. Long-term disability after trauma in children. J Pediatr Surg. 2000;35:684-87.

Van de Voorde P, Sabbe M, Tsonaka R, et al. The long-term outcome after severe trauma of children in Flanders (Belgium): a population-based cohort study using the International Classification of Functioning—related outcome score. Eur J Pediatr. 2011;170:65-73.

Vollrath M, Landolt MA. Personality predicts quality of life in pediatric patients with unintentional injuries: a 1-year follow-up study. J Pediatr Psychol. 2005;30:481-91.

Winthrop A, Brasel K, Stahovic L, et al. Quality of life and functional outcome after pediatric trauma. J Trauma. 2005;58:468-73.

Yacoubovitch J, Lelong N, Cosquer M, et al. Sequelae of Injuries in Adolescents - an Epidemiologic-Study. Arch Pediatr. 1995;2:532-38.
